# A Simple Tenckhoff Catheter Placement Technique for Continuous Ambulatory Peritoneal Dialysis (CAPD) Using the Bandung Method

**DOI:** 10.1155/2020/4547036

**Published:** 2020-06-01

**Authors:** Jonny, Rudi Supriyadi, Rully Roesli, Goh Bak Leong, Lydia Permata Hilman, Fidelisa Cita Arini

**Affiliations:** ^1^Division of Nephrology, Internal Medicine Department, Gatot Soebroto Army Central Hospital, Jakarta, Indonesia; ^2^Division of Nephrology, Internal Medicine Department, Faculty of Medicine, Padjadjaran University/Hasan Sadikin General Hospital, Bandung, Indonesia; ^3^Division of Nephrology, Serdang Hospital, Kajang, Selangor, Malaysia

## Abstract

Insertion of Tenckhoff catheters for continuous ambulatory peritoneal dialysis by nephrologists remains uncommon in most developing countries, including Indonesia. The aim of this study is to describe our experience on a simple technique of Tenckhoff catheter insertion by a nephrologist called the Bandung method. We conducted a retrospective observational study from May 2012 until December 2018 in 230 patients with end-stage renal disease using the Bandung method, a blind percutaneous insertion approach modified from the Seldinger technique. Early complications after insertion were assessed. The mean age of patients was 47.28 years (range 14–84 years). Within 1 month after insertion, complications occurred in 34 patients: 13 (5.7%) malposition, 8 (3.5%) omental trapping, 6 (2.6%) outlow failure, 3 (1.3%) peritonitis, 1 (0.4%) catheter infections, 1 (0.4%) bleeding, 1 (0.4%) kinking, and 1 (0.4%) hernia. None of these complications led to catheter removal. One patient experienced a late (>1 month) post-insertion complication of malposition that could not be repositioned and led to catheter removal. The Bandung method is a simple, cost effective, and minimally invasive technique for Tenckhoff catheter insertion that is associated with the same rate of complications compared to other techniques. This technique may useful for application in developing countries.

## 1. Introduction

The prevalence of end-stage renal disease (ESRD) is increasing. Globally, from 1990 to 2016, the incidence of chronic kidney disease (CKD) increased by 89%, and death due to CKD increased by 98% [1]. This condition increases the need for renal replacement therapy.

Among the various therapeutic modalities for renal replacement therapy, peritoneal dialysis (PD) has several advantages, including more independence, fewer visits to the clinic, much lower doses of erythropoietin [2], greater clearance of higher molecular weight substances than hemodialysis, good control of blood pressure, and unrestricted diet [3]. However, catheter-related complications have significantly limited the long-term effectiveness of continuous ambulatory PD (CAPD) [4].

There are four techniques that are commonly used in the placement of CAPD catheters [5]: open surgical placement [6], peritoneoscopic placement [7], laparoscopic placement [8], and blind placement with Tenckhoff trocar or Seldinger technique with guidewire [9]. The success of CAPD catheters depends on avoidance of three dominant causes of catheter loss: catheter infection, outflow failure, and pericatheter leaks.

The ideal placement method for CAPD catheters is subject to debate. Percutaneous placement is particularly well suited for ailing patients who cannot tolerate general anesthesia. Compared to surgical methods, peritoneoscopy, which provides peritoneoscopic examination of the peritoneal cavity, has lower incidences of fluid leaks and peritonitis and better long-term catheter survival [10]. However, it requires general anesthesia if carbon dioxide is used and is more expensive than blind techniques [11].

The ideal method for the placement of Tenckhoff catheter for CAPD remains debatable. Open surgical and laparoscopic techniques are most frequently used worldwide because of their safety and good initial results. Percutaneous catheter placement (Seldinger technique) is less invasive [10]. Minimizing invasiveness and simplifying techniques are becoming important, particularly in developing countries such as Indonesia.

In the Seldinger technique, the needle is inserted into the cavum pelvis after blunt-dissecting reaches the fascia, and 1-2 L normal saline is administered before inserting the guide-wire. However, this procedure may cause bowel perforation. We have developed a modified Seldinger technique called the Bandung Method. In our technique, the abdominal wall is blunt-dissected until the peritoneum is reached, and the depth of abdominal wall is measured prior to the placement of the introducer needle. By using this technique, bowel perforation and its associated complications can be avoided. The aim of this study is to describe our experience on a simple technique of Tenckhoff catheter insertion by a nephrologist called the Bandung method.

## 2. Materials and Methods

### 2.1. Study Design

The retrospective observational study was conducted at the Division of Nephrology, Internal Medicine Department of Hasan Sadikin General Hospital and Gatot Soebroto Central Army Hospital, Indonesia, from May 2012 to December 2018. The study protocol was reviewed by the institutional review boards. All patients signed an informed approval form for catheter insertion.

### 2.2. Population

ESRD patients scheduled to begin CAPD treatment were included in the study. The patients were selected from those who had no previous abdominal operation or CAPD catheter use.

### 2.3. Technique Description

The CAPD catheter used was a straight, double-cuff, 41 cm Tenckhoff catheter.

All catheters were placed under strict sterile conditions in a semi-intensive room by a nephrologist trained to conduct this procedure on an inpatient basis. All patients fasted for about 8 hours before the procedure and were encouraged to drain their bladder. Bowel preparation was ordered.

During the preinsertion evaluation, coagulation screening was performed, and patients were fully dressed to mark the belt-line location. Catheter selection began with the determination of the catheter-placement site. With the patient in the supine position, the placement site, which coincides with the deep cuff location, was established by aligning the upper border of the catheter with the upper border of the pubic symphysis and by marking the upper border of the deep cuff in the midline, 2 to 3 cm below the umbilicus. The pubic symphysis has been recommended as a reliable marker for the ideal location of the catheter tip in the true pelvis [12].

**Figure 1:**
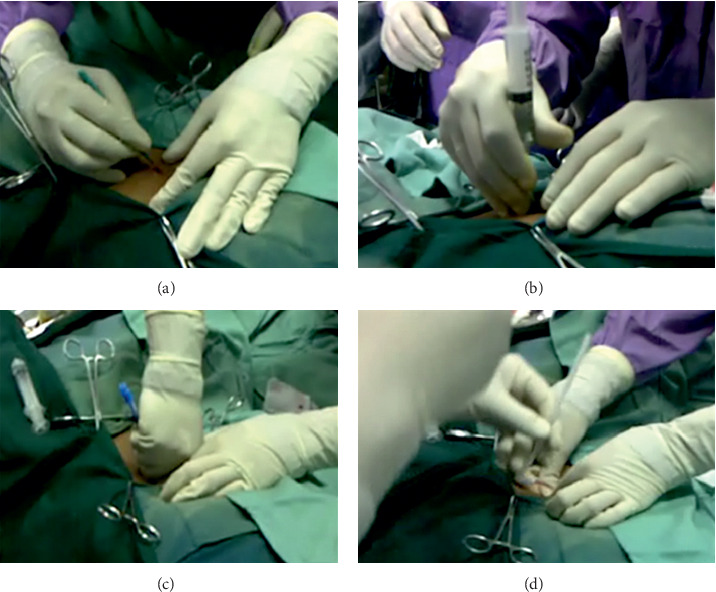
CAPD catheter insertion using the Bandung method. (a) Measurement the depth of the abdominal wall. (b) An introducer needle is implanted toward the pelvis. (c) The dilator with a sheath is inserted over a guidewire. (d) The Tenckhoff catheter is threaded onto a stiffening stylet into the deep pelvis while the sheath is splitting.

Two hours prior to the procedure, an intravenous prophylactic antibiotic was administered (cefotaxime 1 g), and the abdominal wall was shaved. After sterile preparation and draping of the operating area, local anesthesia (lidocaine 2%) was administered by subcutaneous infiltration. A 2.5–3.0 cm horizontal incision was made 2.0-3.0 cm below the umbilicus. The subcutaneous tissue was blunt-dissected up to the peritoneum, and the depth of the abdominal wall was measured (Figure 1(a)). An introducer needle was implanted toward the pelvis (0.5–1.0 cm beyond the depth of the abdominal wall) (Figure 1(b)). Normal saline (20–25 mL) was administered through the introducer needle to ensure that the needle had entered the peritoneal cavity. A guidewire was inserted through the needle into the deep pelvis. The needle was removed, and the dilator with a sheath was inserted over the guidewire (Figure 1(c)). The guidewire and dilator were removed, leaving the sheath in place. The Tenckhoff catheter was threaded onto a stiffening stylet and advanced through the sheath into the deep pelvis while withdrawing the stiffening stylet until the deep cuff reached the peritoneum. The sheath was then split by pulling tabs on both sides (Figure 1(d)). The fascia was sutured such that the deep cuff was overcropped. Using a tunneling stylet, the catheter was directed laterally to the right side, and an exit site was created 2.0-3.0 cm below the placement site. The proximal cuff was placed below the skin, 2.0–4.0 cm from the exit site, and the subumbilical incision was closed.

Fluid in the abdomen was drained. The catheter was irrigated with 500 mL of dialysis solution after insertion. Irrigation was repeated if bleeding was evident after the first irrigation. If the color of the fluid became clear after the second and third irrigations, minor bleeding was considered, and the patient was kept under observation.

Peritoneal dialysis was started on the sixth day with a volume of 2000 mL (4 × 500 mL). If there was no leakage, the PD volume was gradually increased to 8000 mL (4 × 2000 mL) over the ensuing 3 days. The sutures were removed after 1 week. A plain abdominal X-ray was taken a day after procedure.

**Figure 2:**
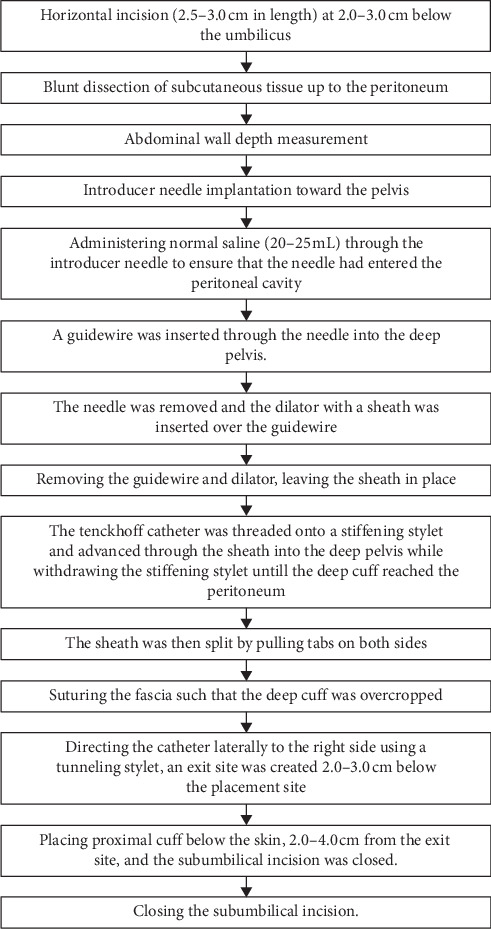
Step-by-step CAPD catheter insertion using the Bandung method.

All patients were followed up; early and late complications were recorded. Early post-insertion complications were defined as those that occurred during the procedure or within 30 days after insertion, and late complications were those that occurred after 30 days [10]. Peritonitis was defined as cloudy effluent with a leukocyte count >100 cells/mL, with more than 50% polymorphonuclear cells. The term “catheter infection” is used to indicate infection in the exit site, tunnel, or both (Figure 2).

## 3. Results

The patients' characteristics are summarized in Table 1. There were 135 males and 95 females with a mean age of 47.28 years (range, 14–84 years). The etiology of ESRD included 74 patients (32.2%) with diabetes mellitus.

The operating time ranged from 30 to 60 minutes (average: 40 minutes). The patients' post-insertion complications are summarized in Table 2. Visceral organ injury was not observed in any patient. There were no instances of pericatheter leakage. One (0.4%) episode of bleeding occurred on the second and third day of care but ceased on the fourth day of care. One (0.4%) patient experienced bilateral inguinal hernia.

There were six (2.6%) cases of outflow failure as an early complication. In these patients, the catheter was repaired by spooling normal saline with guiding ultrasound. Omental trapping occurred in eight (3.5%) patients.

Thirteen (5.7%) episodes of catheter malposition occurred, twelve of which was successfully repaired by replacing the stylet through the catheter. The other catheter (0, 4%) was removed during the third week post-insertion because the malposition could not be repaired. One (0.4%) patient experienced catheter kinking.

Peritonitis occurred in 3 (1.3%) patient. One of the patient's examination revealed the presence of *Mycobacterium tuberculosis* and the catheter was removed during the second week post-insertion. Catheter infection, at the exit site, occurred in 1 (0.4%) patient. The cultures revealed *Staphylococcus aureus* infection, and it was resolved by gentamycin cream application at the exit site.

## 4. Discussion

We developed a percutaneous technique, a modification of the Seldinger technique, which we have called the Bandung method. This procedure allows more direct visualization of the peritoneum and determination of the most suitable site for catheter placement because the proximal cuff can be placed precisely above the peritoneum. The main differences in the steps of PD catheter insertion are briefly summarized in (Table 3). Based on the causes, complications are divided into two groups, infectious and noninfectious. Infectious complications consist of peritonitis, exit-site, and tunnel infections [13] Noninfectious complications comprise catheter dysfunction, dyalisate leakage, hernias, sclerosing encapsulating peritonitis, and bleeding [14].

Pericatheter leakage occurs most frequently during the immediate post-insertion period and is seen in 7%–24% of patients [17]. With the percutaneous technique, lateral placement of the proximal cuff is the preferred method to reduce the incidence of leakage and herniation [18]. A randomized study comparing paramedian versus midline incision revealed that there was no significant difference in the rate of complications or catheter survival [19]. With the Bandung method, there were no instances of pericatheter leakage despite a break-in period of approximately 5 days, with CAPD starting 6 days after catheter placement. Early initiation of PD did not appear to result in more frequent pericatheter leakage. Less pericatheter leakage and a shorter break-in period in the Bandung method might be caused by fascia suturing over rectus abdominis.

Bleeding is a common early complication, especially in patients subjected to long subcutaneous tunneling [20]. Only one episode of bleeding occurred among our patients; however, this resolved by the fourth day of care and the CAPD was initiated on the eighth day of care. This rate is low in comparison with other studies [21, 22]. The bleeding rate using the Bandung method is similar with advanced laparoscopy [23].

Outflow obstruction is a common cause of peritoneal dialysis catheter malfunction. This often occurs because of omental trapping or fibrin obstruction [24]. There were 14 cases of outflow failure (6.1%) in our cohort. Six (2.6%) were caused by fibrin obstruction. In these patients, the obstruction could be repaired by spooling normal saline via ultrasound guidance. Eight (3.5%) were caused by omental trapping. In these patients, the obstruction resolved after laparoscopic omentopexy. The rates of outflow failure is comparable to other techniques [22, 25], while it is higher compared to advanced laparoscopy [26]. A study by Özener et al. did not find significant difference of catheter outcome based on the catheter insertion technique [11]. None of these patients needed catheter removal.

Tenckhoff catheter malposition is one of the leading causes of catheter malfunction [27]. In our cohort, there were 13 episodes of catheter malposition (5.1%). Twelve of which were successfully repaired by replacing the stylet through the catheter. The malposition rate is lower compared to the peritoneoscopic technique [25]. Only one catheter (0.4%) was removed at the third week because the catheter malposition could not be repaired. This rate is lower than the reported for the surgical technique [26].

Peritonitis is the most common cause of CAPD cessation [28]. Bacteria invade the peritoneal cavity via either an intraluminal or a periluminal route. In our cohort, there were three episodes of peritonitis (1.3%) occurred, which is a lower rate than with the peritoneoscopic technique (2.6%) [29] or the open surgery technique (13%) [30]. In our study, the patients who had peritonitis were given antibiotics based on the microbiological test of the effluent, and they continued using CAPD.

*Staphylococcus aureus* is the most common cause of exit-site and tunnel infections [31]. In general, the distal cuff was buried subcutaneously to prevent infection, with the catheter brought through the skin 4–6 weeks later [32]. In our cohort, there was only one (0.4%) instance of exit-site catheter infection. The infection, of *Staphylococcus aureus*, was resolved by applying gentamycin cream on the exit site. These rates are comparable to other techniques [29].

It is not uncommon for abdominal hernia to occur in CAPD patients as the prevalence ranged from 9% to 32%. Anatomically weak sites, uremia, obesity, and poor nutrition due to protein loss, anemia, and the sites of previous abdominal wall surgical procedures are factors that contribute to hernias in PD patients [33]. There was one (0.4%) case of bilateral inguinal hernia in our study. The hernia was treated surgically, and the patient continued PD as dialysis modality. In one study, the incidence of abdominal-wall hernia after catheter placement percutaneously and using conventional surgical technique was 10% and 15.4%, respectively [34]. Though the study observed the complications in a long period of time and our study observed for early complications after catheter placement, the Bandung method have lower rates of hernia. This could happen because our technique keeps the parietal peritoneum intact, thus lowering the probability of hernia, especially incisional hernia.

Kinking can cause outflow failure. The other method is by using metal trochar to straighten the kink under fluoroscopic guidance [35]. Only one episode of kinking occurred among our patients, and it was resolved by using guidewire to straighten the catheter.

Poor catheter positioning during the initial placement is one of major factors that cause early technical failures in CAPD [36]. Using this technique, the complications of catheter placement procedure can be minimized because we can directly visualize the peritoneum and determine the depth of the abdominal wall, thus avoiding bowel perforation. The abdominal wall perforation does not exceed the diameter of the implanted catheter, resulting in elastic sealing of the insertion site. Based on those reasons, PD can safely be initiated earlier (five days after insertion) than the conventional 2–4 weeks. We believe that the risk of bleeding and early catheter leakage may also be reduced through this technique.

The Bandung method is a simple, reliable, minimally invasive technique that does not require an operating room or complicated devices and results in a low rate of complications compared to other techniques. This method will be suitable for use in developing countries, in which the facility for surgery is still limited and for use in archipelago countries, in which access to hemodialysis and kidney transplantation is difficult.

## Figures and Tables

**Table 1:** Patients' characteristics.

Characteristic	*N* (%)
Patients	230
Male : female	135 : 95
Age	
Mean ± SD (years)	47.28 ± 14.94
Range	14–84
Etiology	
DM	74 (32.2%)
Non-DM	156 (67.8%)
Hypertension	65 (28.3%)
Glomerulopathy	56 (24.3%)
Lupus nephritis	21 (9.1%)
Chronic pyelonephritis	9 (3.9%)
Cardiorenal syndrome	1 (0.4%)
Obstructive nephropathy	1 (0.4%)
Polycystic kidney disease	0

SD: standard deviation; DM: diabetes mellitus.

**Table 2:** Complications after insertion.

Complication	*N* (%)
Malposition	13 (5.7%)
Omental trapping	8 (3.5%)
Outflow failure	6 (2.6%)
Peritonitis	3 (1.3%)
Catheter infection	1 (0.4%)
Bleeding	1 (0.4%)
Kinking	1 (0.4%)
Hernia	1 (0.4%)
Pericatheter leakage	0

**Table 3:** Comparison of PD catheter insertion procedure between the Seldinger method and Bandung method [10, 15, 16].

Place of catheter insertion	Seldinger method	Bandung method
	Procedure room of dialysis unit	Procedure room of dialysis unit
Operative procedure:		
Anesthesia	Local ± sedation	Local
Skin incision (location)	Vertical (3–4 cm length)	Horizontal (2.5–3 cm length)
Depth of dissection	Up to fascia	Up to the peritoneum
Administer normal saline before inserting the guide-wire	Yes	No
Depth of introducer needle	Cannot't be predicted	Can be predicted
Suture the fascia to fix deep cuff of Tenckhoff catheter	No	Yes
Break-in period	9 days	5 days

## Data Availability

All relevant data are available within the manuscript.
